# Identification of antisense long noncoding RNAs that function as SINEUPs in human cells

**DOI:** 10.1038/srep33605

**Published:** 2016-09-20

**Authors:** Aleks Schein, Silvia Zucchelli, Sakari Kauppinen, Stefano Gustincich, Piero Carninci

**Affiliations:** 1 Division of Genomic Technologies, RIKEN Center for Life Science Technologies, RIKEN Yokohama Campus, 1-7-22 Suehiro-cho, Tsurumi-ku, Yokohama, Kanagawa 230-0045 Japan; 2 Center for RNA Medicine, Department of Clinical Medicine, Aalborg University Campus Copenhagen, Copenhagen 2450 Denmark; 3 Scuola Internazionale Superiore di Studi Avanzati, Area of Neuroscience, Trieste, Italy; 4 Department of Health Sciences, University of Eastern Piedmont, Novara, Italy; 5 Department of Neuroscience and Brain Technologies, Italian Institute of Technology, Genova, Italy

## Abstract

Mammalian genomes encode numerous natural antisense long noncoding RNAs (lncRNAs) that regulate gene expression. Recently, an antisense lncRNA to mouse Ubiquitin carboxyl-terminal hydrolase L1 (Uchl1) was reported to increase UCHL1 protein synthesis, representing a new functional class of lncRNAs, designated as SINEUPs, for SINE element-containing translation UP-regulators. Here, we show that an antisense lncRNA to the human protein phosphatase 1 regulatory subunit 12A (PPP1R12A), named as R12A-AS1, which overlaps with the 5′ UTR and first coding exon of the PPP1R12A mRNA, functions as a SINEUP, increasing PPP1R12A protein translation in human cells. The SINEUP activity depends on the aforementioned sense-antisense interaction and a free right Alu monomer repeat element at the 3′ end of R12A-AS1. In addition, we identify another human antisense lncRNA with SINEUP activity. Our results demonstrate for the first time that human natural antisense lncRNAs can up-regulate protein translation, suggesting that endogenous SINEUPs may be widespread and present in many mammalian species.

Recent data imply that up to 80% of mammalian genome is transcribed and that many expressed genomic loci produce RNAs from both the sense and antisense DNA strand^1^,^2^,^3^. In fact, more than 50% of all mammalian RNAs may overlap an opposite-strand transcript in a divergent, convergent or a full-length configuration^2^,^4^,^5^,^6^. Antisense lncRNAs, or Natural Antisense Transcripts (NATs) have been shown to regulate gene expression by affecting transcription and mRNA stability^7^,^8^,^9^,^10^. Most NATs were reported to down-regulate their target genes, though selected works also show up-regulation pathways^11^. The mouse SINEUP, Uchl1-AS, was shown to increase translation of the UCHL1 protein in mouse MN9D cells, and relies on a sense/antisense interaction, in which the region of the Uchl1-AS that overlaps with the UCHL1 translation initiation site (TIS) was named as Binding Domain (BD). The SINEUP activity also depends on an inverted SINEB2, subclass 3 retrotransposon embedded within Uchl1-AS, serving as an effector domain (ED)^12^ in the SINEUP. Similarly, the mouse Uxt (Ubiquitously expressed transcript protein) NAT was able to up-regulate the level of the UXT protein, thus representing an additional member of naturally occurring SINEUPs^12^. Using the modular SINEUP structure, synthetic so-called miniSINEUP constructs, comprising a BD, ED and a short spacer sequence, have been generated (Fig. 1a) and shown to efficiently up-regulate recombinant green fluorescent protein (GFP) in HEK293T cells^13^. However, to date no SINEUP homologs have been described in other species.

Here, we identify a NAT to the human protein phosphatase 1 regulatory subunit 12A (PPP1R12A), named as R12A-AS1, and show that it functions as a SINEUP, increasing PPP1R12A protein levels in human cells. Furthermore, we show that a synthetic SINEUP construct, having R12A-AS1 SINE as an ED effectively up-regulates GFP, when combined with a BD targeting the GFP mRNA. Our results demonstrate for the first time that a human NAT can up-regulate protein translation in human cells, suggesting that endogenous SINEUP lncRNAs may be widespread and present in many mammalian species. The short free right Alu monomer repeat element (FRAM) sequence provides a basis for uncovering the molecular mechanism of SINEUP-mediated up-regulation of protein synthesis, and an attractive opportunity for many biotechnological applications.

## Results

### Discovery of NATs with potential SINEUP function in the human brain transcriptome

**Figure 1:**
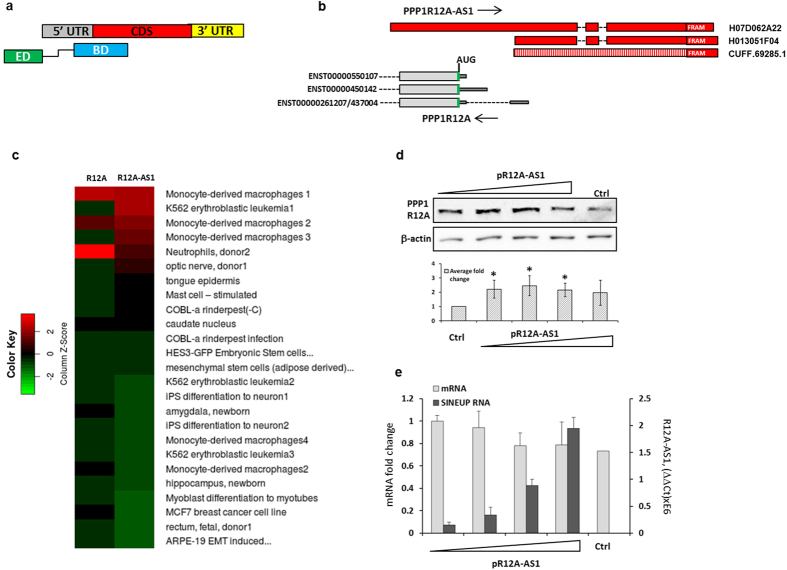
Expression and functional characterization of R12A-AS1. (**a**) Schematic overview of a miniSINEUP and the target mRNA. 5′ and 3′ UTRs, coding sequence (CDS) of the mRNA and the Binding Domain (BD) and Effector Domain (ED) of the SINEUP are indicated. BD and ED are connected with a spacer sequence, (shown as a black elbow line). (**b**) Genomic organization of the PPP1R12A sense-antisense overlapping region (the elements are not in scale). PPP1R12A coding exons are shown as thick grey bars, UTRs as thin grey bars. R12A-AS1 exons in the two RIKEN full-length cDNA clones are shown in red, and introns by dashed lines. Green vertical lines indicate the position of the AUG codon in the mRNA. The R12A-AS1 isoform, assembled by Cufflinks, is shown by a patterned bar, and the position of the FRAM element is indicated by a red box. (**c**) Expression of PPP1R12A and R12A-AS1 in the FANTOM5 dataset. The tpm values for the top 25 samples of Supplementary Table 3 are presented as a matrix plot. (**d**) Western blot analysis of PPP1R12A in HEK293T cells transfected with 30, 60, 150 and 300 pmol of pR12A-AS1, or empty vector as control, respectively. β-actin is shown as a loading control. Lower panel shows mean intensity of PPP1R12A bands, normalized to actin B. (**e**) Corresponding RNA levels, detected by RT-qP PCR (mean ±  s.d. n = 3). *(n = 3, mean + S.D., p < 0.05, One-Sample t-Test, vs empty vector).

The mouse Uchl1-AS is expressed in the brain, which is known to maintain complex and dynamic transcriptional landscapes and contains many noncoding RNAs. Thus, to identify human NATs with possible SINEUP activity, we first generated an inventory of antisense RNAs in the human brain by constructing a cDNA library from brain total RNA for sequencing on the Illumina platform (see Methods section for details). Sequencing reads, which passed initial quality control values (MAPQ ≥ 10, see Supplementary Fig. S1) were assembled into transcripts by Cufflinks^14^,^15^, using RABT-guided assembly pipeline with the RefGene or Ensembl data set as reference annotations. The overview of the processing pipeline is presented in Supplementary Fig. S1. Cufflinks had assembled 1971 RNAs overlapping reference transcripts on the opposite strand. Coordinates of these RNAs were retrieved from Cufflinks’ output and intersected with SINE coordinates, identified in the human genome by Repeat Masker^16^, using Galaxy; a platform for interactive genome analysis at www.usegalaxy.org 17,18. The resulting data set contained 1096 transcripts (Supplementary Fig. S1), overlapping opposite-strand-located mRNAs and at least one SINE. The full list of the transcripts is available as Supplementary Table 1. Next, we intersected these transcripts with coordinates of the last 5 nucleotides of 5′-UTR exons, which come into close contact with the Translation Initiation Site (TIS, usually the first AUG codon of ORFs) on ENSEMBL mRNAs. As a result, 129 transcripts, covering the translation initiation codon of the target mRNA were identified (Supplementary Fig. S1, Supplementary Table 2), and their expression was verified by finding supporting ENCODE/RIKEN CAGE peaks^2^,^19^,^20^ at corresponding loci. In addition, we searched the database of RIKEN full-length human cDNA clones, produced by the Genome Network Project (http://www.osc.riken.jp/english/contents/genom/)^21^ for cDNA clones in these regions. These clone positions are shown in Fig. S1. The figure presents each clone as two separate fragments, because only about 500 nucleotides of the 5′ and 3′ terminal regions had been sequenced, in order to position the clones into the most fitting genomic region. The sequences between the highlighted regions were unknown and have been determined by this work, as indicated in Fig. 1a. To facilitate downstream analysis, factors such as mRNA and protein size and expression level were considered.

### The NAT overlapping protein phosphatase 1 regulatory subunit 12A increases protein translation

We decided to focus our subsequent studies on a NAT to protein phosphatase 1 regulatory subunit 12A (PPP1R12A), because the target protein is expressed at a relatively high level in a wide range of human cells, thus simplifying its detection. The genomic locus of the PPP1R12A 5′ region is shown in Fig. 1b, with a more detailed view with supporting CAGE peaks, clusters and cDNA clones shown in Supplementary Fig. S1. As shown in Fig. 1b, the PPP1R12A mRNA has a number of isoforms, with three possible variants of the 5′ end: a long spliced UTR (ENST00000261207; ENST00000437004), a long unspliced UTR (ENST00000450142) and a short unspliced UTR (ENST00000550107), respectively. The NAT (Fig. 1b), assembled from the brain RNAseq data and named PPP1R12A-AS1 (R12A-AS1), only intersected exon 1 of the long 5′ UTR, leaving the TIS 22 nucleotides upstream (in cDNA coordinates). However, two RIKEN full-length human cDNA clones were also found to intersect the PPP1R12A mRNA in the antisense direction (Fig. 1b). One of these clones, H013051F04 matched almost perfectly the assembled R12A-AS1 RNA, whereas the other, H07D062A22 had its transcription start site (TSS) further upstream, intersecting all known PPP1R12A mRNA isoforms and covering the TIS (Fig. 1b). Both cDNAs ended within a 147 nucleotide-long FRAM, covering 128 (H07D062A22) and 131 (H013051F04) nucleotides of the FRAM, respectively (Fig. 1b). FANTOM5 permissive cluster dataset contained hypothetical TSSs for both variants (Supplementary Fig. S1). Transcription from the plus strand, corresponding to H07D062A22 was additionally confirmed by analysis of the FANTOM5 CAGE dataset^3^ by ZENBU-omics interactive visualization system^22^ at http://fantom.gsc.riken.jp/zenbu/. All samples having tags, mapped to +/−200 nucleotides around H07D062A22′s 5′end (Supplementary Fig. S1) were recorded with their corresponding tag per million reads (tpm) values. The full list of samples and expression values is presented in Supplementary Table 3. As shown in Fig. 1c, blood cells and neural tissues express R12A-AS1 at highest levels, while its restricted expression in specific cell types (Fig. 1b, Supplementary Table 3) suggests a possible regulatory function. Both R12A-AS1 cDNAs were PCR amplified and inserted into the pcDNA3.1 vector, generating the expression constructs pR12A-AS1 (produced from H07D062A22) and pR12A-AS1Δ5′ (produced from H013051F04), respectively.

**Figure 2:**
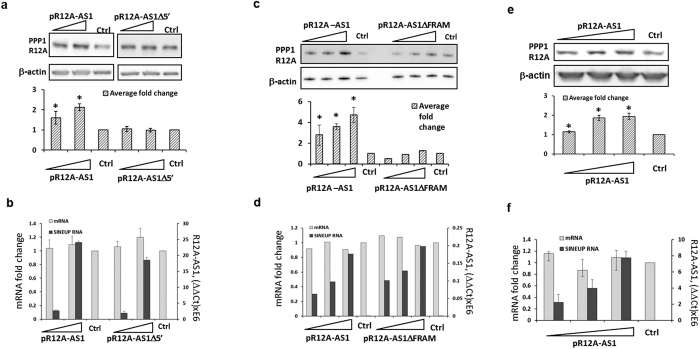
Protein-up-regulating activity of R12A-AS1 depends on the overlap with the mRNA and FRAM. (**a**) Western blot analysis of PPP1R12A in HEK293T cells transfected with the indicated R12A-AS1 clones (60 and 120 pmol) or empty vector as control. Lower panel shows mean PPP1R12A band intensity, as in Fig. 1d. (**b**) RNA levels, as in Fig. 1e. (**c**) Western blot analysis of PPP1R12A in HEK293T cells transfected with 30, 60 and 120 pmol of pR12A-AS1 either without or with the FRAM ED, or empty vector as control. Lower panel shows PPP1R12A band intensity, normalized to actin B. (**d**) The levels of indicated RNAs, detected by qPCR. (**e**) Western blot analysis of PPP1R12A in HeLa cells transfected with pR12A-AS1 or empty vector as control. Lower panel shows PPP1R12A band intensity, as in Fig. 1d. (**f**) RT-qPCR analysis of RNA levels. *(n = 3, mean + S.D., p < 0.05, One-Sample t-Test, vs empty vector).

To ask whether R12A-AS1 functions as a SINEUP, we transfected HEK293T cells with both pR12A-AS1 and pR12A-AS1Δ5′. The level of PPP1R12A protein was analyzed by Western blotting 48 hours after transfection. As shown at Fig. 1d, the protein level was elevated after transfection with pR12A-AS1, without any changes in the PPP1R12A mRNA levels (Fig. 1e). The RNA, produced by pR12A-AS1Δ5′ and only overlapping 5′-UTR did not change PPP1R12A protein level, compared to the full-length RNA (Fig. 2a). This observation confirmed the importance of the TIS-overlapping BD^13^. Notably, deleting the FRAM region from pR12A-AS1 hampered PPP1R12A’s up-regulation (Fig. 2c, lanes 1–3 versus lanes 5–7), consistent with the functional importance of the transposable element (TE) as an ED^13^. Low amounts of the ED-lacking SINEUP construct reduced the PPP1R12A protein levels below control (Fig. 2c, lane 5), which is possibly due to the sense-antisense interaction that could interfere with protein translation in the absence of ED. However, higher amounts of the ED-lacking SINEUP construct restored the protein levels (Fig. 2c, lanes 6 and 7), suggesting that additional sequences within the SINEUP RNA may produce structures, able to mimic the ED.

**Figure 3:**
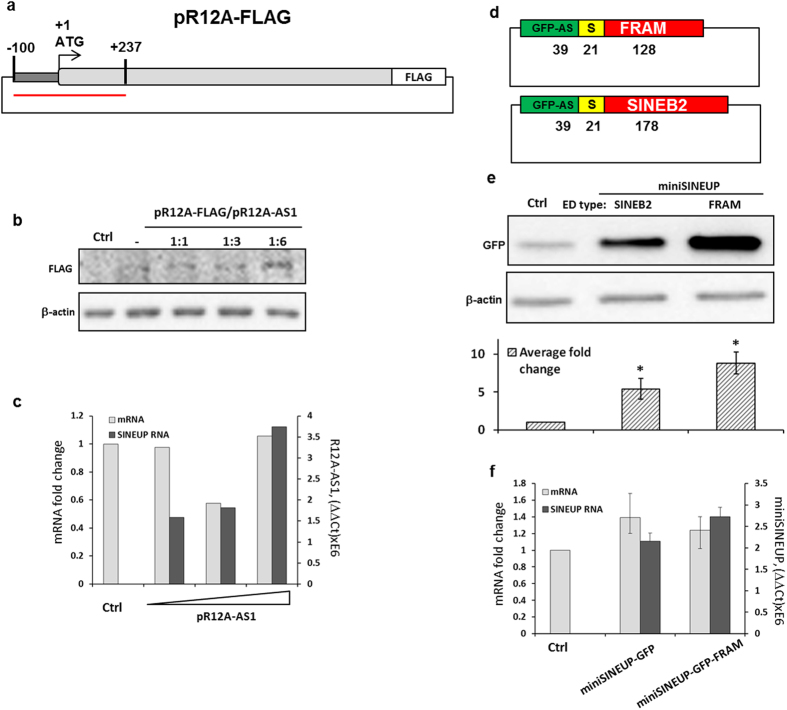
The human SINEUP R12A-AS1 can increase the level of recombinant protein. (**a**) Schematic overview of the FLAG-tagged PPP1R12A construct. Black vertical lines denote the ends of the overlapping region with R12A-AS1. The full overlapping region is highlighted with the red line (**b**) Western blot analysis of the FLAG-tagged PPP1R12A in HEK293T cells, transfected with the R12A-AS1 and pPPP1R12A-F expression constructs, as indicated. (**c,f**) qRT-PCR analysis of PPP1R12A and R12A-AS1 RNAs, extracted from HEK293T cells, in panel (b,e). (**d**) Schematic overview of synthetic miniSINEUPs, containing a FRAM or an inverted SINEB2 element, respectively. The S-region represents the spacer sequence. The length of each sub-element is indicated below (nt). (**e**) Western blot analysis of GFP in HEK293T cells after transfection with the two miniSINEUPs, shown in panel (d). Lower panel shows mean fold change ± s.d. (**f**) RT-qPCR analysis of RNA levels. *(n = 3, mean + S.D., p < 0.05, One-Sample t-Test, vs empty vector).

Similar to HEK293T cells, overexpression of R12A-AS1 in HeLa cells resulted in protein up-regulation (Fig. 2e), without major changes in PPP1R12A mRNA levels (Fig. 2f). In addition, transfection with pR12A-AS1 was able to up-regulate the levels of recombinant, c-terminal-FLAG-tagged PPP1R12A protein in a dose-dependent manner (Fig. 3a–c). The expression of PPP1R12A with 5′ UTR and C-terminal FLAG-tag was consistently very low across all three experiments. However, the up-regulation and the dose response were still observed (Fig. 3b).

### The human FRAM repeat of PPP1R12A-AS1 functions as an ED in a synthetic miniSINEUP

Previously, the SINEB2-derived ED was shown to retain its activity, when transferred to a chimeric antisense RNA, containing the BD for pGFP-c2^12^,^13^. To test whether the human TE sequence is also transferable, we constructed a miniSINEUP^13^, named miniSINEUP-GFP-FRAM with the BD for GFP-c2. Instead of SINEB2, as in the canonical miniSINEUP^13^, it contained the FRAM repeat (Fig. 3d). As shown in Fig. 3e, co-transfection of HEK293T cells with pGFP-c2 and miniSINEUP-GFP-FRAM led to a marked increase of GFP production, without any notable changes in GFP mRNA levels (Fig. 3f). Combined, these findings demonstrate that R12A-AS1 functions as a SINEUP in human cells, and that the FRAM element, when transferred to a synthetic miniSINEUP construct, retains its effect, directed by the sense-antisense overlap, thereby up-regulating heterologous protein production.

### Identification of an additional human NAT with SINEUP activity

**Figure 4:**
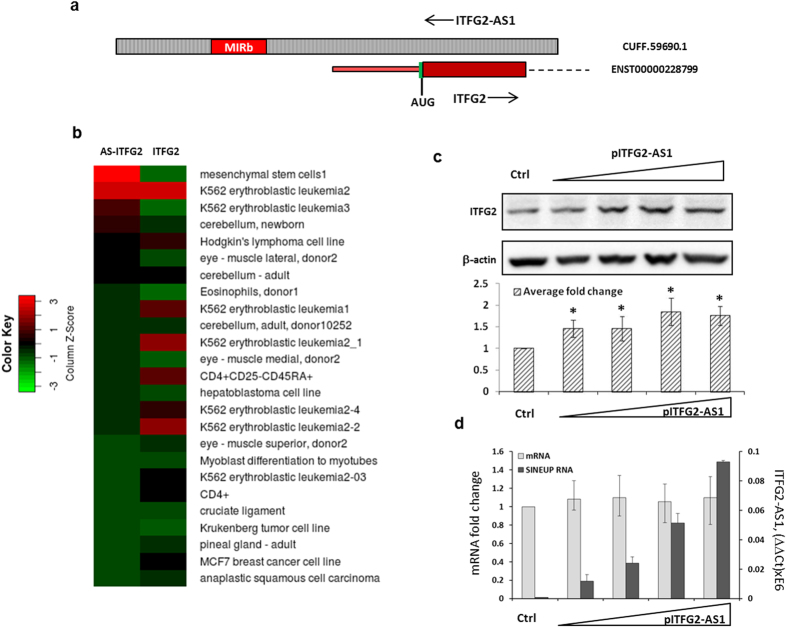
ITFG2-AS1 functions as a SINEUP in human cells. (**a**) Genomic organization of the ITFG2/ITFG2-AS1 overlapping region (the elements are not in scale). The ITFG2 exons are shown in red, ITFG2-AS1 in dashed grey, and introns by dashed lines. Green vertical line indicates the position of the AUG codon in the mRNA. The position of the MIRb transposable element is indicated by a red box. (**b**) Expression of ITFG2 and ITFG2-AS1 in the FANTOM5 dataset. The tpm values for the top 25 samples of Supplementary Table 4 are presented as a matrix plot. (**c**) Western blot analysis of ITFG2 in HEK293T cells transfected with increasing amounts of the pITFG2-AS1 construct or empty vector as control, respectively. β−actin is shown as a control. (**d**) Quantification of ITFG2 mRNA and ITFG2-AS1 RNA levels in the HEK293T cell samples from panel c. *(n = 3, mean + S.D., p < 0.05, One-Sample t-Test, vs empty vector).

Next, we tested a NAT for Integrin-Alpha FG-GAP Repeat-Containing Protein 2 (ITFG2; Supplementary Table 2), another abundant protein, expressed by HEK293T cells for having SINEUP activity. ITFG2 contained an inverted MIRb transposable element as a potential ED. Organization of the ITFG2 genomic locus is summarized in Fig. 4a, and Supplementary Fig. S2. ITFG2 and its antisense RNA are expressed in a broad range of human cells (Fig. 4b and Supplementary Table 4). Similar to R12A-AS1, expression of ITFG2-AS1 in HEK293T cells resulted in up-regulation of ITFG2 at the protein level (Fig. 4c), without affecting mRNA levels (Fig. 4d), implying that also ITFG2-AS1 functions as a SINEUP in human cells.

### Different repeat elements can potentially function as Effector Domains in SINEUPs

Since it is possible that only certain types of repeat elements could function as EDs, we investigated our candidate list of putative human SINEUPs in more detail. The 129 NATs, listed in Supplementary Table 2, contained different SINEs, in almost perfect correlation with their frequencies in the human genome (Pearson correlation coefficient 0.93863). Thus, no enrichment for a given SINE element in the candidate SINEUPs could be identified (Supplementary Fig. S3). The primary sequences of SINEB2 and FRAM originate from different parental genes, tRNA for SINEB2, and 7SL RNA for FRAM, and they do not share any significant sequence similarity, as detected by NCBI BLAST algorithm^23^,^24^. However, a sequence alignment of the two elements using predicted secondary structures (http://www.tcoffee.org/)^25^,^26^, identified short stretches of identical sequences in FRAM and SINEB2, which could potentially fold into similar structures (Supplementary Fig. S4). The use of RNAfold^27^ predicted a long rod-like stem-loop structure for SINEB2, as also previously reported for the mouse SINE^28^, whereas FRAM was predicted to fold into a different structure, comparable to that of 7SL (Supplementary Fig. S5). Thus, more detailed studies are needed to dissect the exact structural requirements of the different types of EDs, which likely share some conserved structural elements that may interact with the translational machinery.

## Discussion

We report here for the first time that two human NATs can function as SINEUPs, and thereby up-regulate protein translation, indicating that this hitherto unknown phenomenon is not only present in mice and men, but could be widespread in a large number of mammalian and possibly other eukaryotic species.

TEs within antisense RNAs may provide binding and recognition sites for molecular factors, regulating translation, while the overlapping antisense region directs the SINEUP activity to specific target sites in mRNAs. Our data on the human FRAM and MIRb TEs reported here suggest that SINEUPs can employ different TEs as their EDs. The short size of FRAM makes it possible to analyze the sequence requirements in more detail, which will be necessary in order to understand the functions of SINEUPs *in vivo*. In addition, a SINEUP RNA, containing FRAM can be readily produced *in vitro*, thus enabling development of new and possibly even shorter SINEUP sequences. RNAs, constructed of minimal ED, short spacer and BD can possibly reach the length of 100–120 nucleotides or less. Molecules of this size are suitable for both viral delivery systems and for use as naked RNAs. Such short and highly efficient synthetic SINEUP RNAs, designed to specifically enhance translation of a given target protein sets the stage for development of SINEUP-based therapeutics for selective up-regulation of protein production for treatment of haploinsufficiencies and metabolic diseases^29^. Both as molecular biology reagents and therapeutic molecules SINEUPs show important advantages over other technologies^29^. Briefly, they increase protein production in the physiological range (2–4-fold), do not modify the cell’s genome and do not overrun cellular regulatory pathways. These features make SINEUPs particularly attractive as tools for research and development of RNA-based therapeutics.

## Methods

### RNA sequencing and bioinformatic analysis

To identify human ncRNA transcripts with possible SineUP activity, we constructed an RNAseq library from 1 ug human brain total RNA, using Epicentre ScriptSeq Complete Kit (#BHMR1205), according to the manufacturer’s instructions. The library was sequenced on the Illumina HiSeq 2000 platform with 100-nt paired-end reads. After splitting sample reads by barcode, eliminating reads mapping to ribosomal DNA and discarding sequences with ambiguous base calling (identified as N), properly paired sequences were mapped to genomes using TopHat v1.4.1 and transcript assemblies were carried out with Cufflinks v1.3.0, using Refseq or Ensembl reference annotation as an optional guide (RABT assembly). Next, we selected transcripts with class code “x”, which overlapped the reference transcripts on the opposite DNA strand. The filtered set of 1971 RNAs was uploaded onto Galaxy public web server (https://usegalaxy.org/). Coordinates of these transcripts were then intersected with SINE repeat coordinates, downloaded from RepeatMasker (http://www.repeatmasker.org), using “Operate on genomic intervals” function in Galaxy. We found that 1096 transcripts out of 1971 overlapped at least one SINE. Finally, these transcripts were intersected with the Ensemble set of reference mRNAs. This analysis reduced the number of candidates to 129 antisense RNAs, containing a repeat element and overlapping with the 5 terminal nucleotides of the mRNA 5′ UTRs.

Alignment of the TE sequences was carried out using RCoffee (http://tcoffee.crg.cat/apps/tcoffee/do:rcoffee), with the default parameters, supplied by the web server. The output was submitted for editing to ESPript 3.0 server directly from the RCoffee result page.

For secondary structure prediction, sequences of FRAM and SINEB2 were submitted to RNAfold web server at http://rna.tbi.univie.ac.at/cgi-bin/RNAfold.cgi, using minimum free energy (MFE) and partition function algorithm.

Quantification of protein levels was performed, using ImageJ Open Source software (http://imagej.net/), according to the program’s manual. The intensity of PPP1R12A bands was normalized to the β-actin levels. The p values were calculated with One-Sample online t-Test Calculator at http://www.danielsoper.com/statcalc/calculator.aspx?id=98. P-values for each significant change in the protein level are provided as the Supplementary Table 6.

### Oligonucleotides

DNA oligonucleotides were ordered from Life Technologies. The complete list of oligonucleotides used for cloning and for quantitative real-time PCR experiments is included in the Supplementary Information (Supplementary Table 5).

### Plasmids

The AS-R12A cDNAs were amplified by PCR from FANTOM clones H07D062A22 and H013051F04, (obtained from DNAForm). AS-ITFG2 cDNA was produced from 1 ug human brain total RNA (Clontech, #636530), using Primescript reverse transcriptase (Clontech, #2680A) and transcript-specific primers. All cDNAs were inserted into pcDNA3.1(-)(Invitrogen) FLAG-tagged cDNAs of PPP1R12A and ITFG2 were produced by a two-step PCR with overlapping forward primers and common reverse primer, containing single FLAG-tag sequence and a stop codon. Only sequences, participating in the sense-antisense interaction were added to the ORFs, based on Supplementary Figs 1 and 4.

The antisense GFP plasmid has been described in Carrieri *et al.*^12^. 39 base pairs corresponding to nucleotide −35/+4 with respect to the ATG of GFP sequence in pEGFP-C2 vector (Clontech) were chosen as target sequence for artificial antisense DNA generation. For TE replacement, SINEB2 was excised with EcoRV and HindIII and substituted by the FRAM sequence produced by PCR.

### Cell culture

Cells were seeded in 100-mm dishes in Dulbecco’s modified Eagle’s (DMEM) medium containing 10% fetal bovine serum (Invitrogen) supplemented with penicillin (50 units ml^−1^) and streptomycin (50 units ml^−1^). For experiments, cells were plated in 6-well plates and grown overnight. Approximately 50% confluent cells were transfected, using Lipofectamine 2000 (Invitrogen) and various amounts of DNA, as indicated in the figure legends.

### qRT–PCR

Total RNA was extracted from cells with RNeasy Mini kit (Qiagen, #74106) according to the manufacturer’s instructions. All RNA samples were subjected to on-column DNase I treatment (Qiagen). A total of 1 μg of RNA was subjected to reverse transcription using PrimeScript First Strand cDNA Synthesis Kit (Takara) and Real Time qRT–PCR was carried out using SYBR^®^ Premix Ex Taq™ (Tli RNaseH Plus) (Takara, RR420A). GAPDH and β-actin were used as normalizing controls in all the other qRT–PCR experiments. The amplified transcripts were quantified using the comparative Ct method and the differences in gene expression were presented as normalized fold expression (ΔΔCt). All the experiments were performed in duplicate. A list of oligonucleotides used for qRT–PCR experiments is shown in Supplementary Table 5.

### Western blot analysis

Cells were resuspended in water and lysed in 2 × SDS sample buffer. Proteins were separated in 10% SDS– polyacrylamide gel and transferred to nitrocellulose membranes. Immunoblotting was performed with the following primary antibodies: anti-PPP1R12A (ab70809 Abcam), anti-ITFG2 (SAB1411084-100UG, Sigma), anti-β-actin (A5441, Sigma). Signals were revealed after incubation with recommended secondary antibodies conjugated with horseradish peroxidase (Daco) by using ECL detection reagent (RPN2105, GE Healthcare). Images were captured by LAS-3000 Imaging System (Fuji) and analyzed. Quantification of protein levels was performed, using ImageJ Open Source software (http://imagej.net/), according to the program’s manual. The intensity of PPP1R12A bands was normalized to the actin B levels. The pvalues were calculated with One-Sample online t-Test Calculator at http://www.danielsoper.com/statcalc/calculator.aspx?id=98.

## Additional Information

**How to cite this article**: Schein, A. *et al.* Identification of antisense long noncoding RNAs that function as SINEUPs in human cells. *Sci. Rep.* **6**, 33605; doi: 10.1038/srep33605 (2016).

## Supplementary Material

Supplementary Information

Supplementary Dataset 1

Supplementary Dataset 2

Supplementary Dataset 3

Supplementary Dataset 4

Supplementary Dataset 5

Supplementary Dataset 6
