# Supplementary material for: Identification of antisense long noncoding RNAs that function as SINEUPs in human cells
Source: Sci Rep. 2016 Sep 20;6:33605. doi: 10.1038/srep33605 (PMC5028707; doi:10.1038/srep33605)
Supplement: Supplementary Information [file srep33605-s1.pdf]

Identification of antisense long noncoding RNAs that function as SINEUPs in human cells

Aleks Schein[1,2], Silvia Zucchelli[3,4], Sakari Kauppinen[2], Stefano Gustincich[3,5] and Piero Carninci[1*]

[1]Division of Genomic Technologies, RIKEN Center for Life Science Technologies, RIKEN Yokohama Campus, 1-7-22 Suehiro-cho, Tsurumi-ku, Yokohama, Kanagawa 230-0045 Japan.

[2]Center for RNA Medicine, Department of Clinical Medicine, Aalborg University Campus Copenhagen, Copenhagen 2450 Denmark

[3]Scuola Internazionale Superiore di Studi Avanzati, Area of Neuroscience, Trieste, Italy

[4]Department of Health Sciences, University of Eastern Piedmont, Novara, Italy

[5]Department of Neuroscience and Brain Technologies, Italian Institute of Technology, Genova, Italy.

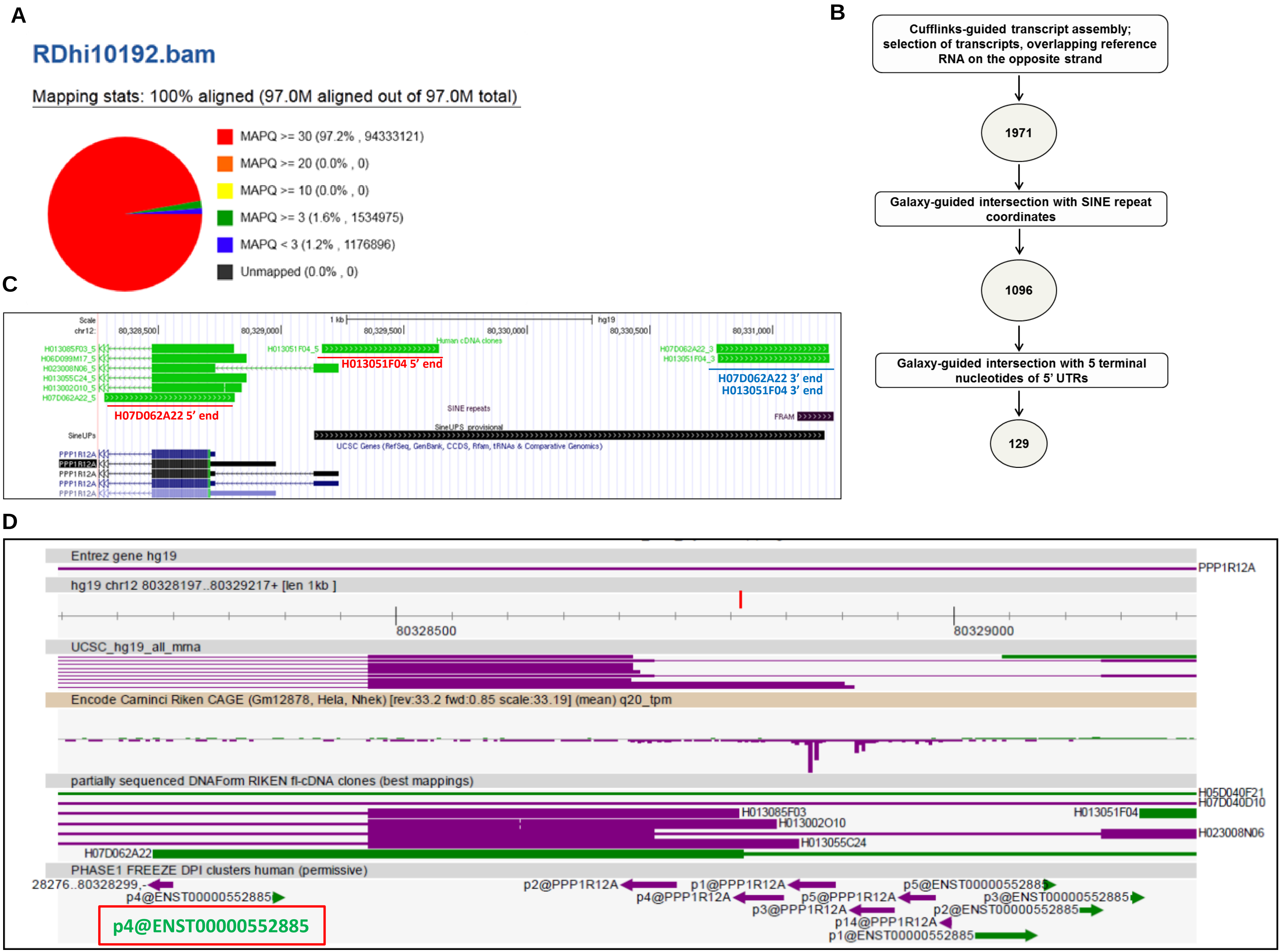

A
RDhi10192.bam
Mapping stats: 100% aligned (97.0M aligned out of 97.0M total)
MAPQ >= 30 (97.2% , 94333121)
MAPQ >= 20 (0.0% , 0)
MAPQ >= 10 (0.0% , 0)
MAPQ >= 3 (1.6% , 1534975)
MAPQ < 3 (1.2% , 1176896)
Unmapped (0.0% , 0)
B
Cufflinks-guided transcript assembly; selection of transcripts, overlapping reference RNA on the opposite strand
1971
Galaxy-guided intersection with SINE repeat coordinates
1096
Galaxy-guided intersection with 5 terminal nucleotides of 5' UTRs
129
C
H013051F04 5' end
H07D062A22 3' end
H013051F04 3' end
H07D062A22 5' end
D
Entrez gene hg19
PPP1R12A
hg19 chr12 80328197..80329217+ [len 1kb]
80328500
80329000
UCSC_hg19_all_mrna
Encode Caminci Riken CAGE (Gm12878, Hela, Nhek) [rev:33.2 fwd:0.85 scale:33.19] (mean) q20_tpm
partially sequenced DNAForm RIKEN fl-cDNA clones (best mappings)
H05D040F21
H07D040D10
H013085F03
H013051F04
H013002O10
H023008N06
H013055C24
H07D062A22
PHASE1 FREEZE DPI clusters human (permissive)
28276..80328299,-
p4@ENST00000552885
p2@PPP1R12A
p1@PPP1R12A
p5@ENST00000552885
p4@PPP1R12A
p5@PPP1R12A
p3@ENST00000552885
p3@PPP1R12A
p2@ENST00000552885
p14@PPP1R12A
p1@ENST00000552885
p4@ENST00000552885

Supplementary Figure S1. Schematic overview of the human SINEUP discovery pipeline.
A. RNAseq library quality control values. Numbers and percentage of mapped reads with specified quality thresholds are shown. **B.** Schematic overview of the pipeline, used for identification of putative human SINEUPs, with the number of transcripts indicated at each step. **C.** UCSC Genome Browser screenshot, showing the 5' region of PPP1R12A. The R12A-AS1 transcript, assembled by Cufflinks is shown as a black bar. The track named "SINE repeats" indicates the position of SINEs, with the repeat name shown near each repeat element. Green bars depict RIKEN full-length cDNA clones, mapped to the locus with clone names shown on the left side. Note that the mapping positions of only ~500 nucleotides from 5' and 3' end are shown. 5' portions are highlighted by red lines, 3'-by blue lines. **D.** ZENBU genome viewer screenshots, showing the 5' region of H07D062A22 cDNA clone (green bar). PPP1R12A mRNA is shown as a set of purple bars. Green arrows indicate the positions of the TSSs for R12A-AS1, TSS, corresponding to the possible start of H07D062A22 is highlighted with the text box. Colors of transcripts and CAGE peaks are show according to the DNA strand: green for positive strand, purple for negative strand.

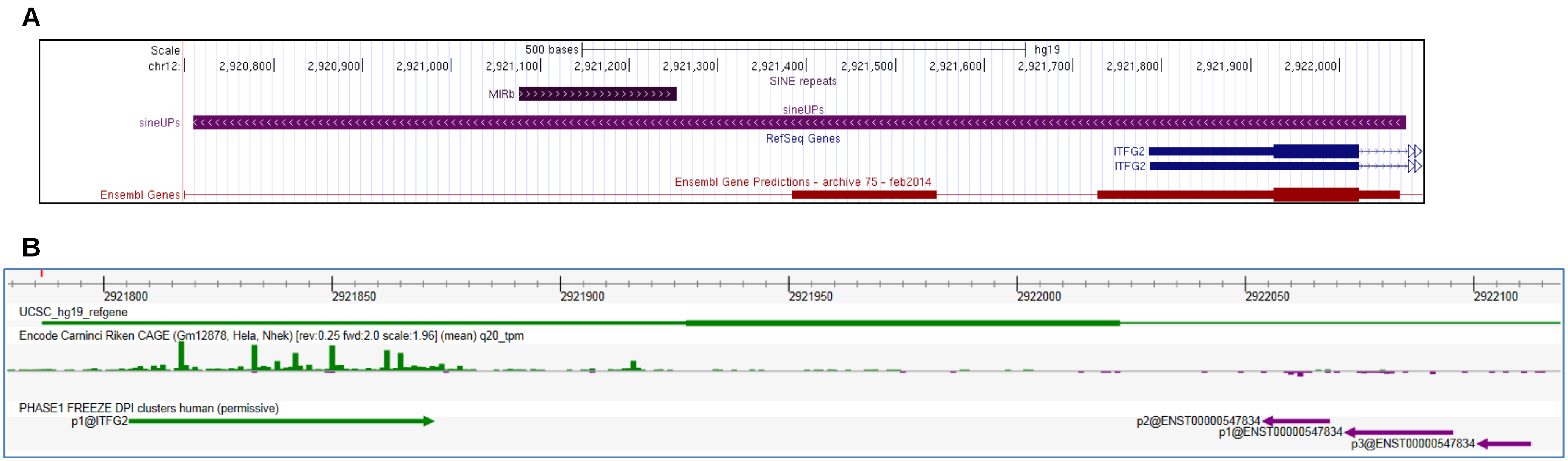

Supplementary Figure S2. Detailed view of the ITFG genomic locus.

a, UCSC Genome Browser screenshot, showing the 5' region of ITFG2. The ITFG2-AS1 transcript, assembled by Cufflinks is shown as a purple bar. Name, location and orientation of SINEs is shown by a black bar. **b,** ZENBU genome viewer screenshots, showing 5' regions of ITFG2 (green bar). Colors of transcripts and CAGE peaks are show according to the DNA strand: green for positive strand, purple for negative strand.

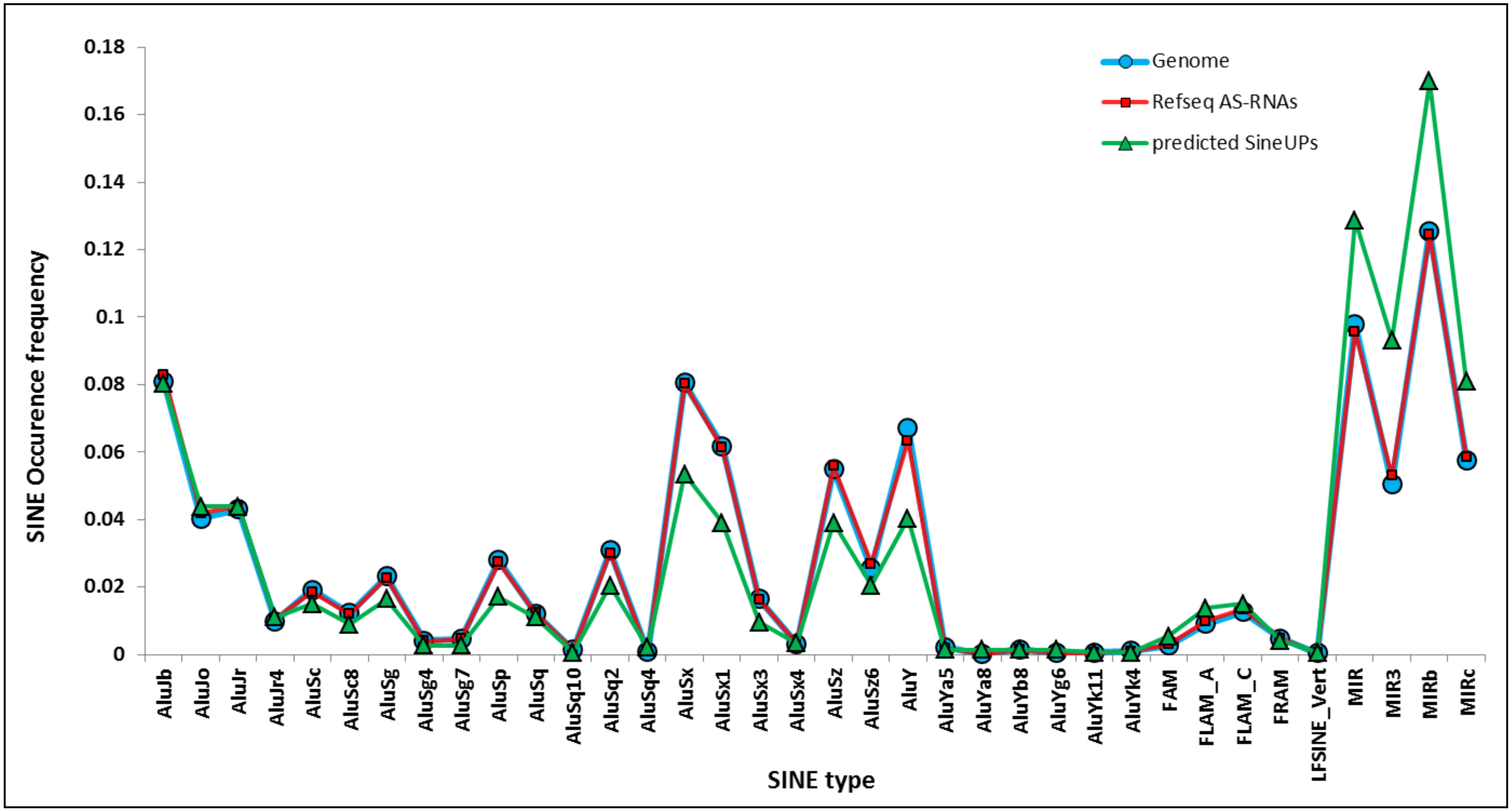

Supplementary Figure S3 NATs, intersecting 5' ends of mRNAs are not enriched for particular types of SINEs. The frequency of each SINE element, present in candidate SINEUP RNAs, listed in Supplementary table 2 (n=1460) was calculated and compared with corresponding general SINE frequency, calculated for the human genome (n=1,793,723) and for antisense RNAs, recorded in the RefSeq database (n=900182). 3 frequencies for each SINE type were plotted on the combined line chart.

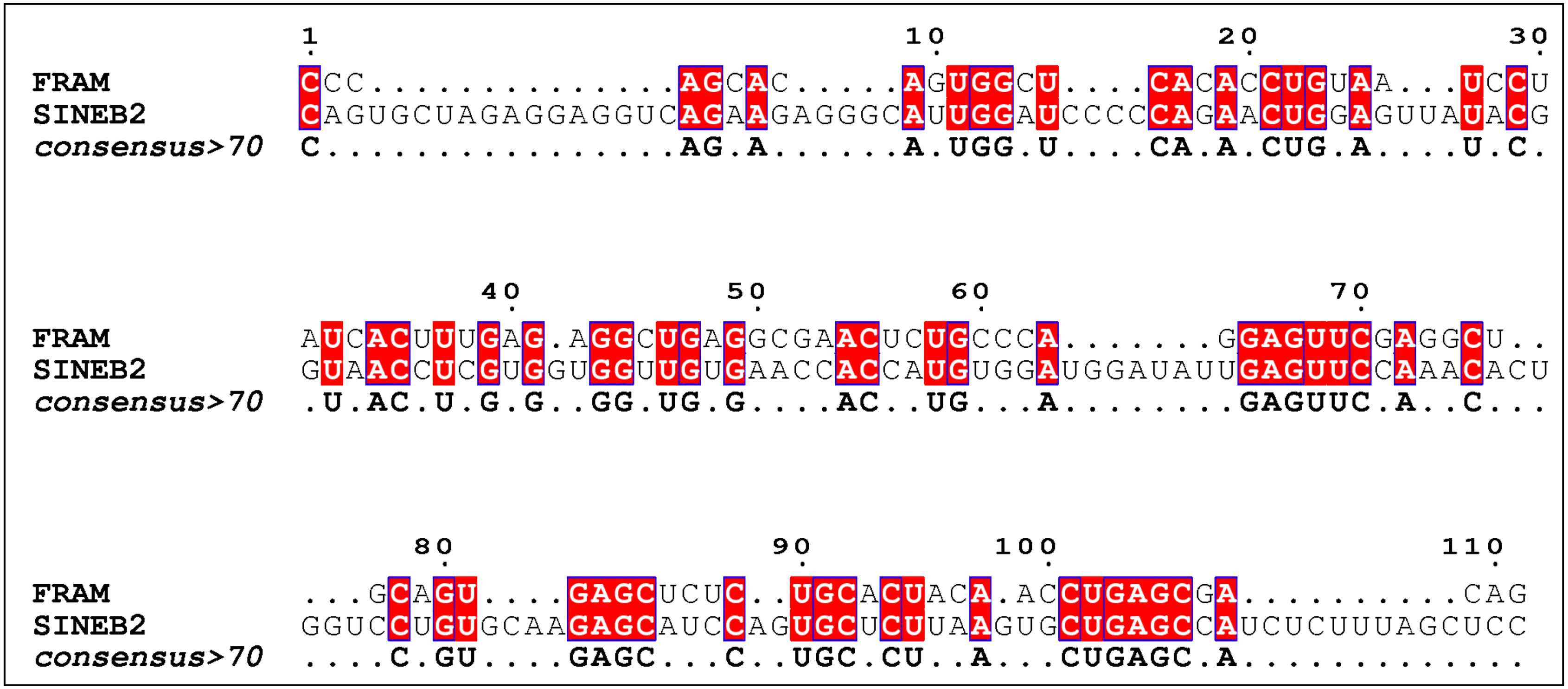

Supplementary Figure S4. Human FRAM and mouse SINEB2 elements share short common sequences .

The SINEB2 and FRAM sequences were analyzed by the R-Coffee software, using default parameters and edited by Espript 3.0.

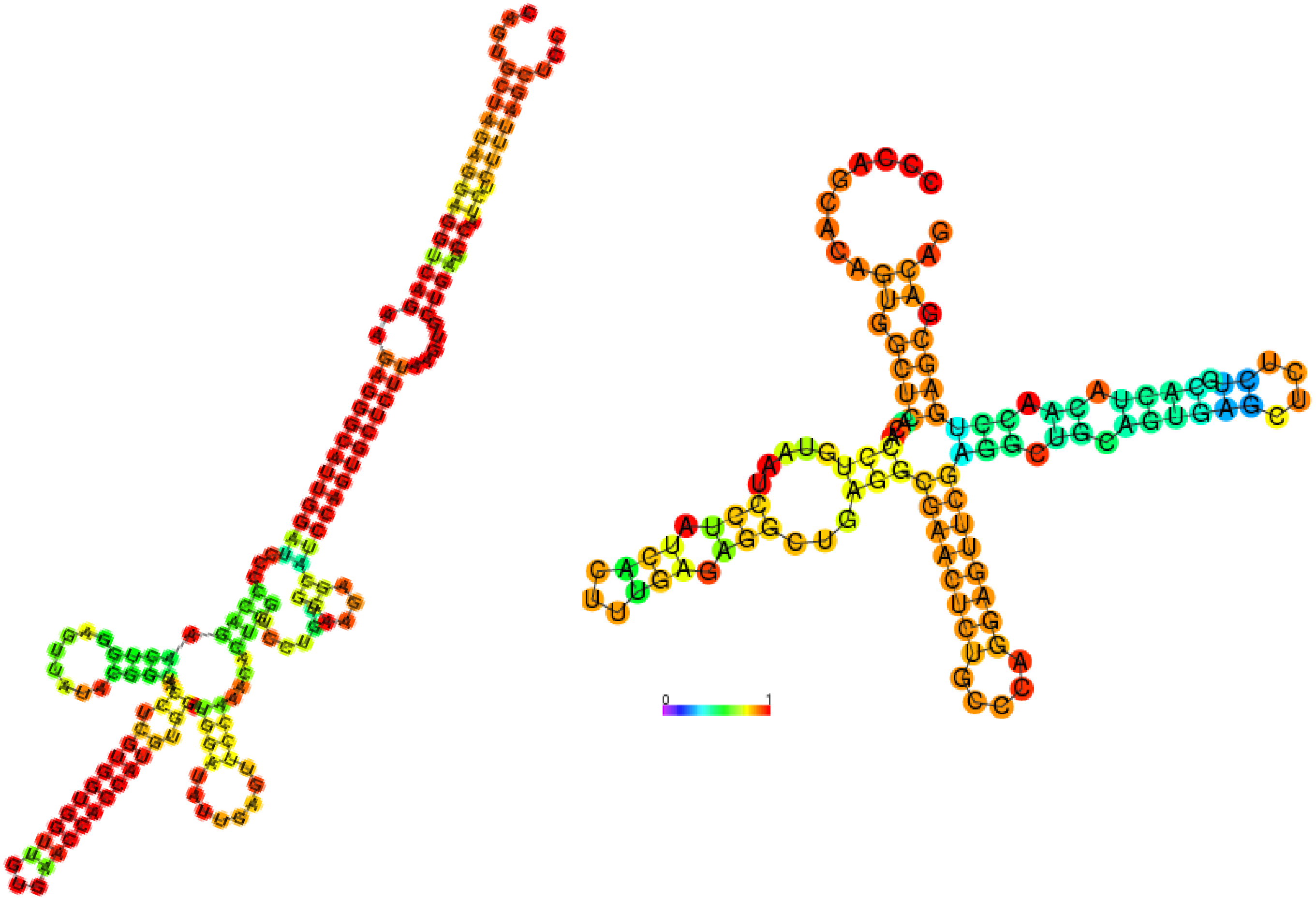

SINEB2 **FRAM**

Supplementary Figure S5. Human FRAM and mouse SINEB2 elements obtain different secondary structures
Secondary structure of SINEs, predicted by RNAfold program. Prediction confidence for each base is indicated by the color code.
